# Supplementary figures and images for: Clinical characteristics and the risk of hospitalization of patients with coronavirus disease 2019 quarantined in a designated hotel in Japan
Source: PLoS One. 2023 Jan 17;18(1):e0280291. doi: 10.1371/journal.pone.0280291 (PMC9844840; doi:10.1371/journal.pone.0280291)

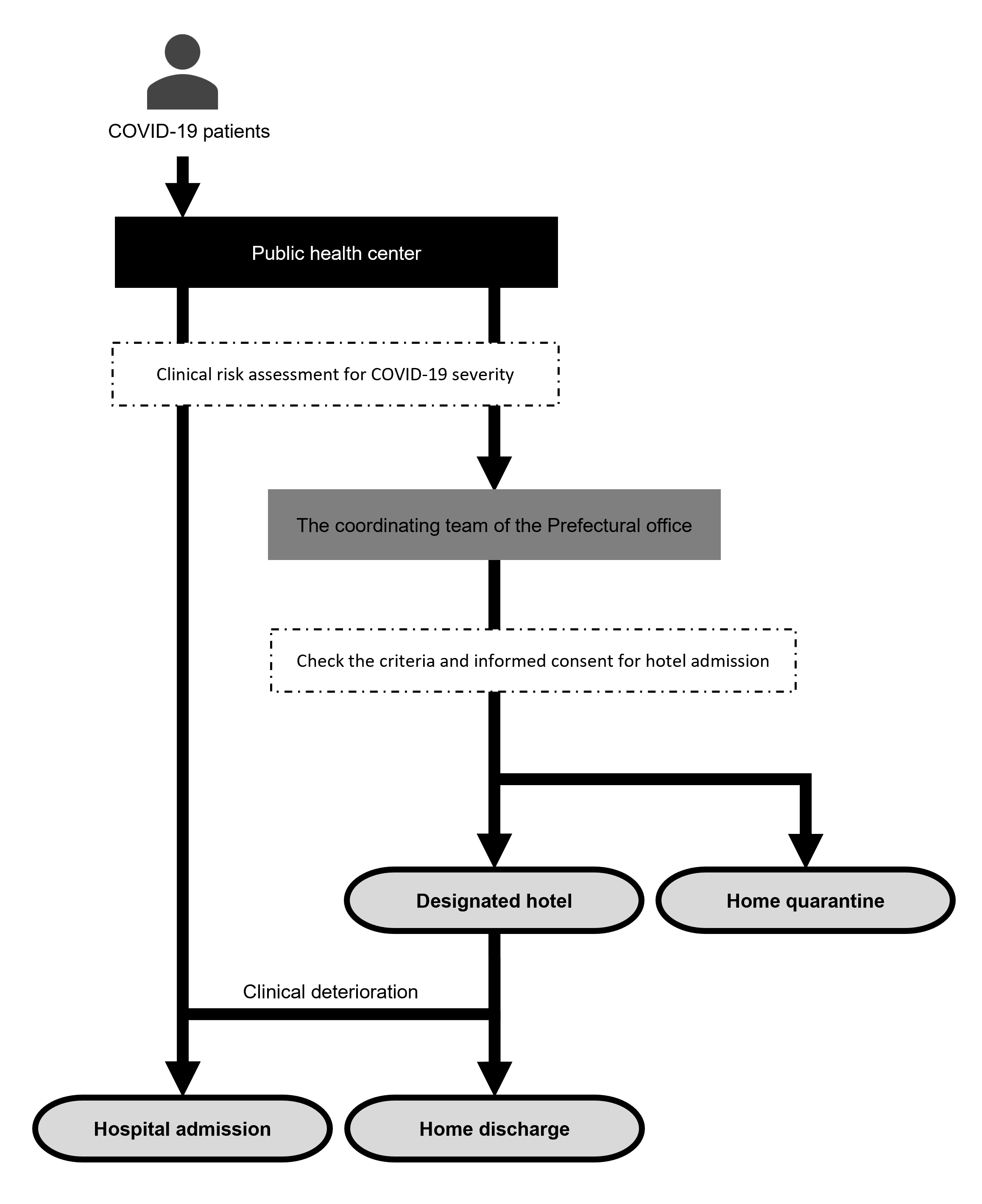

Supplement: S1 Fig — (TIF) [file pone.0280291.s001.tif]
