# Supplementary material for: Clinical characteristics and the risk of hospitalization of patients with coronavirus disease 2019 quarantined in a designated hotel in Japan
Source: PLoS One. 2023 Jan 17;18(1):e0280291. doi: 10.1371/journal.pone.0280291 (PMC9844840; doi:10.1371/journal.pone.0280291)
Supplement: S1 Table — (DOCX) [file pone.0280291.s002.docx]

**S1 Table. Admission criteria and the waves of infection**

|  | 2nd and 3rd waves | 4th wave | 5th wave |
| --- | --- | --- | --- |
| Period | August 2020 to February 2021 | March 2021 to June 2021 | July 2021 to September 2021 |
| Variant | Wild type | Alpha | Delta |
| Number of patients | 114 | 424 | 398 |
| Admission criteria^*1^ | | | |
| Age | Under 40 years | Under 50 years | Under 65 years |
| Severity | Asymptomatic or mild/improving symptoms | Asymptomatic or mild/improving symptoms | Asymptomatic or mild/improving symptoms |
| Respiratory disorder | No respiratory disorder | No respiratory disorder | No oxygen administration required |
| Comorbidities^*2^ | No comorbidities | No comorbidities  (Possible: controlled hypertension with antihypertensive drugs) | No comorbidities (Possible: BMI < 35 kg/m^2^ in case of controlled conditions with appropriate treatments) |
| Immunodeficiency^*3^ | No immunodeficiency | No immunodeficiency | No immunodeficiency |
| Pregnancy | No pregnancy | No pregnancy | No pregnancy |
| Pneumonia | No pneumonia or mild (improving trend) | No pneumonia or mild (improving trend) | No pneumonia or mild (improving trend) |
| Blood test | No remarkable abnormality other than inflammatory reaction | No remarkable abnormality other than inflammatory reaction | No remarkable abnormality other than inflammatory reaction |

*1 There are some discrepancies between the timing of criteria revision and the spread of infection period

*2 Hypertension, diabetes, obesity (BMI >30 kg/m^2^), renal disease, and coronary heart disease

*3 Use of immunosuppressive agent, anticancer agent, and history of organ transplantation
