# Supplementary material for: Clinical characteristics and the risk of hospitalization of patients with coronavirus disease 2019 quarantined in a designated hotel in Japan
Source: PLoS One. 2023 Jan 17;18(1):e0280291. doi: 10.1371/journal.pone.0280291 (PMC9844840; doi:10.1371/journal.pone.0280291)
Supplement: S2 Table — (DOCX) [file pone.0280291.s003.docx]

**S2 Table. Number of infected people reported by each public health center**

| Public health center | Number of patients |
| --- | --- |
| 1. Kuwana public health center | 160 |
| 2. Yokkaichi public health center | 114 |
| 3. Suzuka public health center | 141 |
| 4. Tsu public health center | 198 |
| 5. Matsusaka public health center | 113 |
| 6. Ise public health center | 115 |
| 7. Iga public health center | 74 |
| 8. Owase public health center | 7 |
| 9. Kumano public health center | 7 |
| Outside of the prefecture | 7 |
