# Supplementary material for: Clinical characteristics and the risk of hospitalization of patients with coronavirus disease 2019 quarantined in a designated hotel in Japan
Source: PLoS One. 2023 Jan 17;18(1):e0280291. doi: 10.1371/journal.pone.0280291 (PMC9844840; doi:10.1371/journal.pone.0280291)
Supplement: S3 Table — (DOCX) [file pone.0280291.s004.docx]

**S3 Table. Patients’ characteristics stratified by the period of admission to the hotel**

|  | 2nd/3rd wave  Aug. 2020 – Feb. 2021 | 4th wave  Mar. 2021 – Jun. 2021 | 5th wave  Jul. 2021 – Sep. 2021 | p value |
| --- | --- | --- | --- | --- |
| No. of patients | 114 | 424 | 398 |  |
| Age, years | 23 [20-31] | 26 [21-41] | 32 [22-48] | <0.01 |
| ≤29 years, % | 80 (70.2) | 250 (59.0) | 182 (45.7) | <0.01 |
| 30-39 years, % | 23 (20.2) | 57 (13.4) | 59 (14.8) | 0.20 |
| 40-49 years, % | 8 (7.0) | 80 (18.9) | 72 (18.1) | <0.01 |
| ≥50 years, % | 3 (2.6) | 37 (8.7) | 85 (21.4) | <0.01 |
| Male, % | 68 (59.6) | 247 (58.3) | 259 (65.1) | 0.12 |
| BW, kg | 54.9 ± 8.8^a^ | 62.5 ± 12.2^b^ | 63.0 ± 11.6^c^ | 0.47 |
| BMI, kg/m^2^ | 21.4 ± 3.1^a^ | 22.2 ± 3.2^d^ | 22.5 ± 3.4^c^ | 0.08 |
| <18.5 kg/m^2^, % | 3 (100.0)^a^ | 27 (9.9)^d^ | 43 (10.8)^c^ | <0.01 |
| 18.5-25.0 kg/m^2^, % | 0 (0.0)^a^ | 189 (69.5)^d^ | 268 (67.5)^c^ | 0.04 |
| ≥25.0 kg/m^2^ | 0 (0.0)^a^ | 56 (20.6)^d^ | 86 (21.7)^c^ | 0.63 |
| Comorbidities |  |  |  |  |
| Hypertension | 1 (0.9) | 14 (3.3) | 28 (7.0) | <0.01 |
| Diabetes mellitus | 0 (0.0) | 1 (0.2) | 0 (0.0) | 1.00 |
| Dyslipidemia | 0 (0.0) | 8 (1.9) | 10 (2.5) | 0.23 |
| Hyperuricemia | 0 (0.0) | 7 (1.7) | 6 (1.3) | 0.50 |
| Malignancy (including history), % | 0 (0.0) | 2 (0.5) | 3 (0.8) | 0.83 |
| Asthma (including history), % | 0 (0.0) | 7 (1.7) | 10 (2.5) | 0.21 |
| Smoking, % | 4 (3.5) | 91 (21.5) | 112 (28.1) | <0.01 |
| Temperature on hotel admission, °C | 36.5 ± 0.4 | 36.7 ± 0.5 | 36.8 ± 0.6 | <0.01 |
| Temperature ≥ 37.0 °C on hotel admission, % | 19 (16.7) | 132 (31.1) | 139 (34.9) | <0.01 |
| SpO_2_ on room air at hotel admission, % | 98.4 ± 0.7 | 97.8 ± 0.8 | 98.7 ± 1.3 | <0.01 |
| SpO_2_ on room air at hotel admission ≥ 97%, % | 13 (11.4) | 114 (26.9) | 77 (19.3) | <0.01 |
| Symptoms during clinical course | | | | |
| Asymptomatic, % | 25 (21.9) | 35 (8.3) | 7 (1.8) | <0.01 |
| Fever (≥ 37.5 °C), % | 24 (21.1) | 291 (68.6) | 355 (89.2) | <0.01 |
| High-grade fever (≥ 38.5 °C) | 1 (0.9) | 27 (6.4) | 63 (16.2) | <0.01 |
| Max temperature, °C | 37.0 ± 0.4 | 37.2 ± 0.6 | 37.5 ± 0.8 | <0.01 |
| Cough, % | 33 (28.9) | 198 (46.7) | 259 (65.1) | <0.01 |
| Shortness of breath, % | 6 (5.3) | 15 (3.5) | 16 (4.0) | 0.70 |
| Sore throat, % | 23 (20.2) | 189 (44.6) | 226 (56.8) | <0.01 |
| Runny nose/nasal obstruction, % | 50 (43.9) | 151 (35.6) | 138 (34.7) | 0.19 |
| Olfactory dysfunction, % | 49 (43.0) | 50 (11.8) | 55 (13.8) | <0.01 |
| Dysgeusia, % | 43 (37.7) | 59 (13.9) | 49 (12.3) | <0.01 |
| Fatigue, % | 31 (15.3) | 188 (42.9) | 223 (54.3) | <0.01 |
| Loss of appetite, % | 2 (1.8) | 5 (1.2) | 28 (7.0) | <0.01 |
| Headache, % | 11 (9.6) | 161 (38.0) | 152 (38.2) | <0.01 |
| Nausea/Vomiting, % | 5 (4.4) | 3 (0.7) | 20 (5.0) | <0.01 |
| Diarrhea, % | 7 (6.1) | 32 (7.5) | 66 (16.6) | <0.01 |
| Arthralgia/Myalgia, % | 3 (2.6) | 76 (17.9) | 99 (24.9) | <0.01 |
| Hospitalization due to clinical deterioration | 6 (5.3) | 18 (4.2) | 28 (7.0) | 0.22 |

BW: body weight; BMI: body mass index; SpO_2_ percutaneous oxygen saturation

^a^N=3, ^b^N=245, ^c^N=397, ^d^N=272
