# Supplementary material for: Clinical characteristics and the risk of hospitalization of patients with coronavirus disease 2019 quarantined in a designated hotel in Japan
Source: PLoS One. 2023 Jan 17;18(1):e0280291. doi: 10.1371/journal.pone.0280291 (PMC9844840; doi:10.1371/journal.pone.0280291)
Supplement: Attachment — Submitted filename: Response to Reviewers.docx [file pone.0280291.s005.docx]

Response to reviewer:

Thank you very much for your suggestions. Your comments were very useful for improving our manuscript. According to your and the other reviewers’ comments, we have updated our manuscript, **Table 1, Table 2, Figure 4,** and **S3 Table**.

Major Comments

**1.**	I'm afraid the study concerning an isolation facility in Tokyo, Japan, has already been published. (Sakamoto, et al. PMID: 35717438) Since the sample size of this study was smaller than that of the previous research, it is necessary to show the strengths of this study compared to the previous research. The authors need to focus on the novelty of this study.

**RESPONSE:**

Thank you for your important suggestion. As you pointed out, the results from Sakamoto et al. were similar to those of our research. However, 1) this is the first report to investigate the clinical characteristics of COVID-19 patients who were admitted to a designated hotel in rural a prefecture, and 2) to present the novel risk score to identify the high-risk patients based on simple parameters including age, BMI, body temperature and SpO_2_.

We added the reference you provided, and revised the sentence in the introduction and discussion section as follows:

[Introduction]

**“Therefore, the purpose of the present study was to investigate the clinical characteristics of COVID-19 patients who were admitted to a designated hotel, and to clarify** **the safety of the designated hotel accommodation program and** **identify the risk factors for hospitalization during outbreaks or pandemic periods using the medical records in Mie Prefecture, Japan.” (Page 3, Line 57 – Page 4, Line 60)**

[Discussion]

**“**The present study ~~is the first to have~~ investigated the safety of the designated hotel accommodation program and identified risk factors for hospitalization in patients with COVID-19 in Japan.**” (Page 18, Line 268–270)**

**“**… and 3) the new **validated** 7-point risk scale successfully identified high-risk patients for hospitalization.**” (Page 18, Line 273)**

**“****Recently,** **Sakamoto et al. has reported the clinical characteristics of COVID-19 patients who were admitted to public accommodation facilities and risk factors for hospitalization in metropolitan Tokyo** **[****Y Sakamoto, et al. J Infect Chemother. 2022;28:1439-1444.].** **In their research, 6.4% of patients were hospitalized, comparable to the present result (5.9%). Although the accommodation program was managed by each local government, the hospitalization rates were similar between rural prefectures and Tokyo metropolitan areas. Furthermore,** no fatal case was observed in the designated accommodation program during hotel stays in Mie Prefecture.**” (Page 20, Line 301-307)**

**“****Sakamoto et al. reported that age over 40 years and BMI ≥ 25 kg/m^2^ were the risks for hospitalization in metropolitan Tokyo [Y Sakamoto, et al. J Infect Chemother. 2022;28:1439-1444.].** **Importantly, the present study presented a novel risk score to identify the high-risk patients based on simple parameters including age, BMI, body temperature and SpO_2_. Since body temperature and SpO_2_ are essential elements in the management of respiratory infections, this novel risk scoring system can be a reliable method to identify COVID-19 patients at high risk of hospitalization.” (Page 23, Line 359-364)**

**2.**	The prediction model's method is not clearly stated. In other words, the method of making the predictive model seemed somewhat arbitrary: one of my concerns is the cutoff value (e.g., age of 37 and BMI of 24), and the other concern is the fitting of the model.

How did you decide on the fitting of the model? Did you collect only those that were clinically significant or only those that were statistically significant? Further mention would be needed on this point. If this point cannot be addressed, the study should be revised, focusing on a descriptive study.

**RESPONSE**

Thank you for your important suggestion. We agree that the method in the original manuscript was poorly documented. At first, we identified the independent predictors of hospitalization using a logistic regression analysis by adjusting for clinically relevant factors, such as general background characteristics, comorbidities, and vital sign data with p values < 0.01 on univariate analysis (Please see New **Table 2**). Second, the optimal cut-off values of age, BMI, temperature, and SpO_2_ were investigated using ROC curve analyses (Please see S2 Figure). Third, we created new risk scale based on the results of adjusted odds ratios of binary logistic regression analysis.

We added New **Table 2,** revised **Table 3** and revised the sentence in the method section as follows.

**“L**ogistic regression analysis was also **performed** to investigate the independent predictors of hospitalization, which was constructed by adjusting for clinically relevant factors, such as general background characteristics, comorbidities, and vital sign data with p values < 0.01 on univariate analysis**. Then, the optimal cut-off values of the identified independent predictors of hospitalization such as** age, BMI, temperature, and SpO_2_ were investigated using ROC curve analyses (S2 Figure). The odds ratio (OR) of each clinical risk factor for hospitalization was calculated by **binary** logistic regression analysis adjusted by age, male sex, BMI, hypertension, temperature at hotel admission, and SpO2 at hotel admission. **A new risk scale based on the results of adjusted odds ratios was created.” (Page 9, Line 160-168)**

**“Table 3 shows that o**lder age (≥ 37 years), higher BMI (≥ 24 kg/m2), higher temperature (≥ 37.0 °C), and lower SpO2 (≤ 97%) on hotel admission were the independent prognostic factors for hospitalization.**” (Page 12, Line 223-225)**

[Table legend]

**Table 2. Predictors of hospitalization on logistic regression analysis**

**3.**	Since the model did not do the external validation, the model had severe limitations. Could the authors perform external validations using another dataset or boot-strap method?

**RESPONSE**

We appreciate the reviewer’s comment of this point. According to your advice, we performed internal validation using Boot-strap method. Our scoring model also had good discrimination (C-index, 0.8561) and calibration (Hosmer–Lemeshow test χ2 = 40.500, P value = 0.1958). These statistical analyses were performed using R (version 4.2.1).

We added **Figure 4** and revised the sentence in the method and results section as follows.

[Methods]

**“Internal validation of the original data was performed using the bootstrap method with 200 repetitions of sampling to evaluate whether the scoring system could accurately predict the risk of hospitalization.** Significance was defined as a p value < 0.05, and all statistical analyses were performed using EZR (version **1.60**), which is a graphical user interface for R (version **4.2.1**).**” (Page 9, Line 171 - Page 10, Line 174)**

[Results]

**“The bootstrap method was used to internally validate the risk scoring, and the calibration curve was plotted after the original data were repeatedly sampled 200 times (Fig 4). The corrected C-index, calibration intercept, and calibration slope were 0.8561, 0.0294, and 1.0042, respectively. After bootstrap calibration, the mean absolute error was 0.009, and the Hosmer-Lemeshow goodness-of-fit test χ2 = 40.500, P = 0.1958, indicating that the model had good prediction accuracy.” (Page 17, Line 245-249)**

[Figure legend]

**Fig 4. Calibration curve with bootstrap resampling validation for predicting the risk of hospitalization.**

Mainor Comments

**1.**	Regarding Table S1, did the all patients take blood or radiographic tests before the hotel admission?

**RESPONSE**

Thank you for your important suggestion.

**“Hotel admission was permitted if the physician decided that the blood and radiographic examinations were not needed. Indeed, almost all patients (96.5%) who were admitted from their home did not undergo blood or radiographic examinations before the hotel admission.”**

We added the above sentences in the section of the method. **(Page 5, Line 87-90)**

**2.**	Were the patients with mental disorders included in the hotel-quarantine patients? If so, could that information (having mental illnesses or not) be added to the covariates?

**RESPONSE**

Thank you for your important suggestion. Unfortunately, there were no detailed data on mental disorders for each patient before hotel admission in the present study.

**3.**	As for the criteria of discharge from the quarantine hotel, did it need "COMPLETE" improvement of the patient's symptoms? This seems to differ from the criteria generally used in Japan. In Japan, the end of isolation requires that “respiratory symptoms are improving.” (<https://www.mhlw.go.jp/stf/covid-19/qa.html>)

**RESPONSE**

We apologize for confused you. We revised the incorrect description as follows.

**“**The patients could be discharged from the hotel 10 days after the onset of symptoms and **48** hours after **the resolution of fever or respiratory** symptoms had **improved.” (Page 7, Line 126-127)**

**4.**	Although the authors said "does not provide medical treatment (line 75)", the authors also described "the doctor could also prescribe oral medications for the patients if necessary. (line 117)". This seems to be confusing for readers outside Japan. The authors should state that "face-to-face medical care, including oxygen administration and intravenous fluid therapy, could not be provided" and "however, telemedicine, including drug prescription (without anti-viral drugs), could be provided."

**REPONSE**

Thank you for your valuable suggestions on our manuscript. We revised the manuscript according to your suggestion.

**“**~~Since the designated hotel is a non-medical facility and does not provide medical treatment,~~ **D**octors and nurses were dispatched from the cooperating medical institutions, including Mie University Hospital, and they were stationed at the hotel to monitor patients’ conditions and to respond to sudden deterioration. If the stationed doctors or nurses determined that **a** patient**’s** symptoms or condition had worsened, the coordinating team of the prefectural office quickly arranged for hospital treatment (S1 Figure). **” (Page 4, Line 74 – Page 5, Line 79)**

**“The designated hotel is a non-medical facility, and, therefore, face-to-face medical care including oxygen administration and intravenous fluid therapy could not be provided. However, if a patient described symptoms or requested a medical consultation, the on-call doctor examined the patient by telephone or video. Thus, telemedicine, including drug prescription (without anti-viral drugs), could be provided.” (Page 7, Line 120-124)**

**5.**	Were all patients with fever lasting more than 2 (consecutive?) days transported and admitted to the hospital? Or did medical providers "consider" transportation? This difference may be significant because of selection bias. The authors should describe it in detail.

**RESPONSE**

We completely agree with your suggestion. In our research, all patients with persistent fever (≥ 38.5 ℃ more than consecutive 2 days) were transported and admitted to the hospital. Then, we revised the manuscript as follows.

“…, if the patient developed sudden onset dyspnea, oxygen desaturation (SpO2 < 93%), chest pain, orthopnea, wheezing, difficulty breathing, ill complexion, persistent fever (≥ 38.5 ℃ more than 2 **consecutive** days), inadequate oral intake (less than 30% for more than 2 days), difficulty walking, or wound/fractures that required treatment. **All patients who met the above criteria were transferred to the hospital.” (Page 7, Line 136-138)**

**6.**	Was the age of patients normally distributed? If not, the authors should change the notation using median and IQR.

**RESPONSE**

Thank you for your suggestion. As you pointed out, the age is non-normally distributed data. We revised **Table 1**, New **S4 Table**, and the manuscript as follows.

**“Non-normally distributed data are reported as medians [interquartile range (IQR)].” (Page 9, Line 154-155)**

**7.**	Although about half of the Japanese people had been vaccinated as of September 2021, the number of vaccinated patients in this cohort was too small. Could the authors explain the reason?

**RESPONSE**

Thank you for your important suggestion. Until June 2021, the number of vaccinated people was low. Indeed, Sakamoto, et al. reported that the vaccinated patients who were admitted to the recovery accommodation facilities was also very low [Y Sakamoto, et al. J Infect Chemother. 2022;28:1439-1444.] (no patients were vaccinated between November 2020 and March 2021, 0.1% were vaccinated between April 2021 and May 2021, and 11.8% were vaccinated between June 2021 and July 2021. However, as you pointed out, the vaccinated rate in our study cohort was lower than expected. We revised the manuscript on this point as follows.

**“****In Japan, the vaccination rate was low until June 2021, and it dramatically increased to about 50% by September 2021. The number of vaccinated patients who were admitted to a designated hotel between August 2020 and September 2021 was very small in the present cohort, presumably because of the low vaccination rate and the high effectiveness of vaccination in the younger population.” (Page 18, Line 276-279)**

**8.**	The authors should include the waves of COVID-19 surge in the covariate. This is because the characteristics and pathogen of the variants of SARS-CoV-2 (e.g., alpha variant, delta variant…) differed. For the authors, it may be challenging to identify the SARS-CoV-2 variant of the eligible patients. The waves of COVID-19 should be a surrogate covariate for the SARS-CoV-2 variants.

**RESPONSE**

Thank you for your important suggestion. Unfortunately, surveillance tests to identify SARS-CoV-2 variants were not performed in all cases. Furthermore, as shown in S1 Table, the admission criteria were revised at every wave of COVID-19 infection in Mie prefecture. Based on the results, we did not use the waves of COVID-19 as a surrogate covariate for the SARS-CoV-2 variants. We added the below sentences in the limitations section.

**“Fourth, surveillance tests to identify SARS-CoV-2 variants were not performed in all cases, though the characteristics and the variants of SARS-CoV-2 differed. Furthermore, the admission criteria were revised with every wave of COVID-19 infection in Mie prefecture. Thus, the waves of COVID-19 were not used as a surrogate covariate for the SARS-CoV-2 variants.” (Page 24, Line 387-391)**

**9.**	Could the authors show the reference of the sentence in Lines 279-281 in terms of privacy protection? The readers outside Japan may wonder how the authors knew this information.

**RESPONSE**

Thank you for your suggestion. According to your suggestion, we added the reference, Iwamoto S. Man with mild COVID-19 found in a state of cardiac arrest. The Asahi Shimbun. 2020 December 12 [Cited 2022 October 19]. Available from: https://www.asahi.com/ajw/articles/14013991.

Removed:

“Sudden deaths were also reported in Tokyo, Saitama, Osaka, and Hyogo Prefecture.” **(Page 310, Line 310-311)**

**10.**	The authors should discuss the difference between isolation in hotels and isolation at home. For example, their families monitored the patients staying in their homes. While the patients isolated in the hotels might die without anyone noticing because the room in the hotel does not have a security camera and because the patients communicate with someone only by the telephone or some SNS tool. This may imply home quarantine may be safer than hotel isolation. Could the authors show the merit of hotel isolation using the data in this study?

**RESPONSE**

Thank you for the thoughtful comment. This point is very important, however, we don’t have the data of home isolation, and could not evaluate the differences between isolation in the hotel and isolation at home. So, we added the sentence to the limitation section as follows.

**“****Fifth, the data of home isolation were not available, and it was not possible to evaluate the differences in safety between hotel accommodation and home isolation. If families or roommates monitored the patients isolated at home, home quarantine may be safer than hotel isolation.” (Page 24, Line 391 – Page 25, Line 393)**

**11.**	In Line 335, the sentence, "The findings might suggest that the timing of care escalation was too late," would be an intuitive leap. If the authors' opinion was correct, an overwhelming number of patients must have been transported to the hospitals, and as a result, the healthcare system would collapse.

**RESPONSE**

We agree with your suggestion. According to your suggestion, we removed the sentence.
