# Supplementary material for: Clinical characteristics and the risk of hospitalization of patients with coronavirus disease 2019 quarantined in a designated hotel in Japan
Source: PLoS One. 2023 Jan 17;18(1):e0280291. doi: 10.1371/journal.pone.0280291 (PMC9844840; doi:10.1371/journal.pone.0280291)
Supplement: Attachment — Submitted filename: Response to Reviewers.docx [file pone.0280291.s006.docx]

Response to reviewer:

Thank you very much for your suggestions. Your comments were very useful for improving our manuscript. According to your comments, we have updated our manuscript, **Table 1, Table 2, Table 3, Figure 3,** and **Figure 4**.

**1.**	Initially, I thought that the novelty of this study was in the description of the patient characteristics who received treatments at public quarantine accommodations in Mie Prefecture. However, if the authors think that the main subject of this study is to assess the risk factors for hospitalization from the facilities, the authors and I need to discuss this further.

(1) Since patients are admitted to the hospital if their body temperature (BT) is over 38.5°C for more than two days, BT ≥37.0, one of the risk factors, seems to be an intermediate variable (not a confounding factor) of admission. In other words, it is obvious that patients with high body temperature will be transferred and admitted.

SpO2 may also be an intermediate factor; however, we can also interpret that SpO2 may be a confounding factor because hypoxia of hotel-admitted patients was derived from COPD or other lung diseases. The authors should reconsider whether these are the appropriate risk factors as well.

The authors should provide references that indicate that such intermediate variables are allowed to be included in the prediction model, or the authors should remove these vital signs from the prediction model.

**RESPONSE:**

As you pointed out, body temperature and SpO_2_ seem to be an intermediate variable of hospitalization. We removed these variables from the regression analysis.

We revised **Table 2, Table 3, Figure 3, Figure 4,** and sentence as follows.

[Abstract]

“The logistic regression analyses showed that older age (≥ **40** years), higher body mass index (≥ **25** kg/m^2^), **and presence of hypertension** were the independent risk factors for hospitalization. As the new risk scale score based on the results of the odds ratios increased, the hospitalization rate increased significantly: **2.0**% at 0-1 points, **9.7**% at 2-3 points, and **28.8**% at 4-5 points (p < 0.001).” **(Page 2, Line 30 – 35)**

[Methods]

“**Logistic regression analysis was also performed to investigate the independent predictors of hospitalization, and odds ratio (OR) of each clinical risk factor for hospitalization was calculated. ” (Page 9, Line 160 – 168)**

“**A simple risk scoring system was created using the significant hospitalization factors. Points were assigned to correlate the score with the ORs determined by the logistic regression model,** and the hospitalization rate was stratified by the number of scale points.” **(Page 9, Line 169 – Page 10, Line 173)**

[Results]

“**We created a simple scale based on the results of the logistic regression model, and classified patients based on their score on this scale as follows: Group 1 (score 0-1, 69.4%), Group 2 (score 2-3, 24.3%), and Group 3 (score 4-5, 6.3%) (Table 3).** **Fig** **3** shows that the hospitalization rate according to the risk scale of COVID-19 severity identified in this study. As the new risk scale score increased, the hospitalization rate increased significantly: **2.0% at 0-1 points, 9.7% at 2-3 points, and 28.8% at 4-5 points (p < 0.001)**. The bootstrap method was used to internally validate the risk scoring, and the calibration curve was plotted after the original data were repeatedly sampled 200 times (Fig 4). The corrected C-index, calibration intercept, and calibration slope were **0.7612, 0.0412, and 1.0084**, respectively. After bootstrap calibration, the mean absolute error was 0.009, and the Hosmer-Lemeshow goodness-of-fit test **χ2 = 44.739, P = 0.5806**, indicating that the model had good prediction accuracy.” (**Page 13, Line 240 – Page 14, Line 250)**

[Discussion]

“The major findings were: 1) around 6% of patients were hospitalized, but none of them died or developed serious complications from COVID-19; 2) older age, higher BMI, and **presence of hypertension** were the risk factors for hospitalization due to clinical deterioration; and 3) the new validated risk scale successfully identified high-risk patients for hospitalization.” (**Page 19, Line 299 – 304)**

Removed:

“The present study showed that older age, higher BMI, higher temperature, and lower SpO_2_ were the independent risk factors associated with deterioration and hospitalization. However, the cut-off values of any risks were milder in the current cohort population than in previous studies. These results may be mainly due to differences in race or ethnicity and the hotel admission criteria. At the start of hotel recuperation, the hotel admission criteria were set based on the following criteria: under 40 years of age, asymptomatic or mild/improving symptoms, no preexisting comorbidities, and BMI ≤ 30 kg/m^2^.” (**Page 23, Line 375 – 381)**

[Conclusions]

“Age ≥ **40** years, BMI ≥ **25** kg/m^2^, and **presence of hypertension** were **the** risk factors for hospitalization, and the new risk score successfully identified the higher risk patients.” **(Page 26, Line 429 - 432)**

(2) If the above risk factors are not included in the prediction model, the authors cannot show novelty compared with the previous studies.

This is a personal opinion, though; the fact that the population density is different from that of previous studies may lead to the novelty of this study.

Specifically, the novelty could be expressed by focusing on the difference between Tokyo, a densely populated area, and Mie, a relatively sparsely populated area.

However, this comment can be substantially different from the authors' thoughts, so if the authors have a good idea, please discard this opinion of mine.

**RESPONSE:**

Thank you for your valuable suggestions on our manuscript. We revised the manuscript in the discussion section according to your suggestion.

“Although the accommodation program was managed by each local government, **the hospitalization rates were similar between a relatively sparsely populated rural prefectures and a densely populated metropolitan Tokyo areas.**” **(Page 21, Line 335 – 337)**

(3) Can the age be categorized? If the authors want to create a scoring system, they should divide the population into 3-4 categories of age and score them according to odds ratios. This may show the difference from the previous studies.

**RESPONSE:**

Thank you for your important suggestion. We evaluated the risk of hospitalization by stratified age and BMI. Furthermore, we scored them according to the odds ratios.

We revised **Table 1**, **Table 3** and the sentence as follows.

[Results]

**“The odds ratio increased with age and was significantly higher among patients aged > 50 years (OR = 14.2, 95% confidence interval: 6.18-32.6) than that in the other age groups. Presence of hypertension and obesity were also associated with hospitalization.” (Page 13, Line 236 - 239)**

[Figure legend]

**“Fig 3. The hospitalization rate according to the risk scale score of COVID-19 severity identified in this study. The risk scale is shown in Table 3. Statistical analyses were performed with Fisher’s exact test, followed by Holm’s post hoc test for multiple comparisons. *p < 0.001 vs 0-1 points; † p < 0.001 vs 0-1 points and 2-3 points.”**

**2.**	I could not understand why you did not include N waves of COVID-19 patient surge as a covariate.

If the admission criteria were changed depending on the waves, it is even more likely to be a confounding factor of the outcomes and should be included in the covariate.

I believe that regarding the waves of surge as covariates are not sufficient for addressing the confounding. If the authors consider that the heterogeneity of the patients in each wave is stronger, it is necessary to stratify the calculation of odds ratios by each wave.

At the very least, the fact that "different waves have different criteria for hospitalization" is not a reason to "not add waves to the covariate."

**RESPONSE**

Thank you for your important suggestion. We agree with your opinion. We calculated the odds ratios using the logistic regression model.

We revised **Table 2** and revised the sentence as follows.

[Results]

**“The rates of hospitalization were not significantly changed among the waves of COVID-19 patient surge.” (Page 13, Line 235 - 236)**

[Limitations]

Removed:

“Furthermore, the admission criteria were revised with every wave of COVID-19 infection in Mie prefecture. Thus, the waves of COVID-19 were not used as a surrogate covariate for the SARS-CoV-2 variants.” **(Page 25, Line 419 – Line 421)**

We moved the following sentence to **Page 12, Line** **222**.

“S3 Table presents the patients’ characteristics divided into three groups according to the waves of COVID-19 infections. Due to the revision of the admission criteria, the age of patients of the 5th wave was higher, and they had a significantly higher prevalence of comorbidities (especially hypertension) compared to the those of the 2nd/3rd wave or 4th wave. Some symptoms, such as fever, cough, sore throat, and arthralgia/myalgia were more frequent in the patients of the 5th wave than of the 2nd/3rd wave or 4th wave, but there was no significant difference in the hospitalization rates.”
